# Assessing Electronic Health Literacy in Individuals With the Post–COVID-19 Condition Using the German Revised eHealth Literacy Scale: Validation Study

**DOI:** 10.2196/52189

**Published:** 2024-04-25

**Authors:** Matthias Marsall, Hannah Dinse, Julia Schröder, Eva-Maria Skoda, Martin Teufel, Alexander Bäuerle

**Affiliations:** 1 Institute for Patient Safety University Hospital Bonn Bonn Germany; 2 Clinic for Psychosomatic Medicine and Psychotherapy LVR–University Hospital Essen University of Duisburg-Essen Essen Germany; 3 Center for Translational Neuro- and Behavioral Sciences University of Duisburg-Essen Essen Germany

**Keywords:** eHealth literacy, eHEALS, factor analysis, measurement invariance, psychometric properties, infodemic

## Abstract

**Background:**

The eHealth Literacy Scale (eHEALS) is a widely used instrument for measuring eHealth literacy (eHL). However, little is known so far about whether the instrument is valid for the assessment of eHL in persons who are affected by the post–COVID-19 condition. This is particularly important as people with the post–COVID-19 condition are frequently affected by false information from the internet.

**Objective:**

The objective of our study was to evaluate the validity and reliability of the German Revised eHealth Literacy Scale (GR-eHEALS) in individuals with the post–COVID-19 condition.

**Methods:**

A cross-sectional study was conducted from January to May 2022. The self-assessment survey consisted of the GR-eHEALS, health status– and internet use–related variables, sociodemographic data, and (post)–COVID-19–related medical data. Confirmatory factor analysis (CFA), correlational analyses, and tests of measurement invariance were deployed.

**Results:**

In total, 330 participants were included in the statistical analyses. CFA revealed that the 2-factor model reached an excellent model fit (comparative fit index=1.00, Tucker–Lewis index=0.99, root mean square error of approximation=0.036, standardized root mean square residual=0.038). Convergent validity was confirmed by significant positive correlations between eHL and knowledge of internet-based health promotion programs, experience in using these programs, and the duration of private internet use. In addition, a significantly negative relationship of eHL with internet anxiety supported convergent validity. Further, significant relationships of eHL with mental health status and internal health locus of control confirmed the criterion validity of the instrument. However, relationships of eHL with physical health status and quality of life could not be confirmed. The 2-factor model was fully measurement invariant regarding gender. Regarding age and educational level, partial measurement invariance was confirmed. The subscales as well as the overall GR-eHEALS reached good-to-excellent reliability (Cronbach α≥.86).

**Conclusions:**

The GR-eHEALS is a reliable and largely valid instrument for assessing eHL in individuals with the post–COVID-19 condition. Measurement invariance regarding gender was fully confirmed and allows the interpretation of group differences. Regarding age and educational level, group differences should be interpreted with caution. Given the high likelihood that individuals with the post–COVID-19 condition will be confronted with misinformation on the Internet, eHL is a core competency that is highly relevant in this context, in both research and clinical practice. Therefore, future research should also explore alternative instruments to capture eHL to overcome shortcomings in the validity of the GR-eHEALS.

## Introduction

In the current phase of the COVID-19 pandemic, it has become apparent that infection with SARS-CoV-2 can be associated with the experience of prolonged physical and cognitive impairments. According to the clinical case definition by the World Health Organization, the occurrence of specific delayed symptoms about 12 weeks after recovery from COVID-19 infection is referred to as the “post–COVID-19 condition” and is estimated to affect about 10%-35% of people with infection [1,2]. Individuals with this condition regularly report physical and psychological symptoms, such as fatigue, muscular weakness, and sleep difficulties, as well as anxiety and depression [3]. Current data from Germany demonstrate that employees on sick leave due to the post–COVID-19 condition were unable to work for an average of 90 days. Moreover, people who needed inpatient treatment more than 7 days due to the post–COVID-19 condition were unable to work for an average of 168 days [4]. Data from Sweden likewise confirmed high incidences of about 13% of individuals with the post–COVID-19 condition resulting in long sick leave periods [5]. Nevertheless, studies have also reported that employees, especially health care workers, return to work despite ongoing symptoms due to the post–COVID-19 condition, which could be associated with negative long-term health effects [5,6]. These findings imply that the post–COVID-19 condition is a major public health issue with a significant impact on entire societies and public health systems. However, the etiology of the post–COVID-19 condition is not conclusively understood, and psychosocial factors may have a significant impact on physical and cognitive impairments experienced after COVID-19 infection [7,8].

In addition to the illness-related burden of the post–COVID-19 condition, individuals affected are faced with a high amount of false or confusing information, mostly spread via social media [9]. This comprises all topics around the COVID-19 pandemic, including symptoms, medication, treatments, and vaccination, as well as denial of the existence of the virus and the disease at all [10-12]. In this context, it has become apparent that individuals with COVID-19–related symptoms are susceptible to encountering misinformation, referred to as the COVID-19 “infodemic” [10,13-16]. This infodemic arose right after the beginning of the worldwide pandemic, and an analysis to investigate the proportion of false information revealed that more than 80% of the information spread via social media was presumably wrong [17]. Education about the existence of COVID-19–related misinformation and the labeling of misinformation may be relevant factors in maintaining a functioning health care system [10,18].

The fact that individuals are confronted with false or dubious health information from the internet is not a new phenomenon [19]. The concept of the competence in dealing with health information from the internet—eHealth Literacy (eHL)—was introduced back in 2006 by Norman and Skinner [20]. The concept and its measurement were based on the assumption that individuals may have competencies to seek, find, understand, and evaluate health information from the internet and to distinguish valid and reliable health information from dubious or wrong information [20]. Namely, eHL comprises peoples’ skills, knowledge, and competencies required to navigate, evaluate, and effectively use health information from digital sources, such as websites, apps, and online communication platforms, to make informed decisions about health-related issues and engage in self-care, health promotion, and disease prevention activities [20-22]. The measurement instrument developed by the same researchers, the eHealth Literacy Scale (eHEALS) [23], was translated into several languages and validated in many countries worldwide [24-33]. Over the years of research regarding eHL, studies have shown that higher eHL is associated with health-related outcomes, such as better health behaviors and health cognitions in older adults [34,35], more regular sports exercises in students [36], better health behaviors regarding physical exercise and eating behavior in adults [21], and higher physical and mental health in patients after percutaneous coronary intervention [37]. Moreover, studies have shown that individuals with higher eHL show higher adherence to prevention behavior guidelines in the context of the COVID-19 pandemic [38,39]. Vaccination is a critical factor in reducing the infection rates and severity of COVID-19 symptoms and therefore the burden on the public health system [40]. Consequently, initiatives to improve eHL could increase acceptance of vaccinations as part of a human-centered strategy to reduce the burden on the health care system [41].

In summary, humans benefit from high eHL, which empowers them to effectively use health information from the internet to cope with physical or psychological impairments. Due to the high density of information, and especially due to the high proportion of false information regarding COVID-19, people with COVID-19 or the post–COVID-19 condition have particularly high requirements for well-developed eHL. Because when people are exposed to the fact that around 80% of health information is not evidence based, it is even more difficult to identify the correct and evidence-based information from the internet and apply it to their own health situation [17]. In addition, the use of social media for obtaining health information has changed significantly during the COVID-19 pandemic [42,43]. More precisely, people obtain health information more frequently from social media sources rather than from homepages. This has fundamentally changed the way health information is obtained, which raises the question of whether eHEALS is still adequate to capture the concept of digital health literacy in the changed information era. In addition, criticism regarding the measurement is also arising with regard to the consistency of health-related outcomes as well as the actuality of eHEALS in an increasingly networked, digital environment [44-48]. Nevertheless, eHEALS is, to date, the most widely used instrument to assess eHL [44].

Therefore, a validation of eHEALS is needed to investigate the applicability of this assessment in terms of validity and reliability in a changed environment for people with the post–COVID-19 condition. In this study, we focused on the application of eHEALS in measuring eHL in German-speaking individuals with the post–COVID-19 condition. Specifically, this study aimed to fill the following research gaps:

- First, we evaluated the construct validity and reliability of the German version of eHEALS, the German Revised eHealth Literacy Scale (GR-eHEALS) [49], in a sample of individuals with the post–COVID-19 condition (aim 1).
- Second, we investigated the convergent validity of the GR-eHEALS (aim 2).
- Third, we examined the criterion validity of the GR-eHEALS (aim 3).
- Fourth, we tested the equivalence of the measurement properties of the GR-eHEALS regarding sociodemographic variables of individuals with the post–COVID-19 condition as part of the construct validity (aim 4).

## Methods

### Ethical Considerations

Before taking part in the survey, all participants received study information. Electronic informed consent was digitally obtained at the beginning of the survey from all participants. There was no compensation for participation in the study. Due to the anonymous study design, no individual information of participants is reported in this paper. The study was executed in accordance with the Declaration of Helsinki and was approved by the Ethics Committee of the Medical Faculty of the University of Duisburg-Essen (19-89-47-BO).

### Study Design and Participants

A cross-sectional online survey study was conducted. Data were collected via the online survey system Unipark (Tivian XI GmbH). Participation in the survey was anonymous, voluntary, and without monetary compensation. The participants of this study were recruited between January and May 2022. Flyer and information materials were laid out in different hospitals (eg, University Hospital Essen) and rehabilitation clinics (eg, MEDIAN clinics) in North Rhine-Westphalia, Germany. Furthermore, self-help communities on different social media platforms (eg, Facebook) were contacted, and online flyers were distributed. To participate in this study, the following inclusion criteria were applied: age≥18 years, internet access, a good command of the German language, confirmed COVID-19 infection in the past, and reporting as currently having post–COVID-19 symptoms. COVID-19 infection was assessed via a self-report of the date of a positive detection of SARS-CoV-2 with a nasopharyngeal swab. Symptoms related to the post–COVID-19 condition were assessed according to the clinical case definition by the World Health Organization [2]. For this purpose, participants were asked about the presence of the following symptoms: sore throat, cough, shortness of breath, headache/pain in the limbs, body temperature above 38°C, olfactory or gustatory disturbances, diarrhea, and other symptoms that could be entered via a text field. Multimedia Appendix 1 shows how frequently each of these symptoms was reported.

Participants took on average 13 (SD 5.43) minutes to complete the survey. Participants were able to stop the survey at any time. 554 people have participated in the study. According to the inclusion criteria, we excluded n=196 (35.4%) participants who reported that they currently have no post–COVID-19 symptoms. Further, we excluded participants with implausible responses regarding age (age<18 years: n=3, 0.5%; age>100: n=1, 0.2%) and outliers regarding the survey completion time (n=24, 4.3%). The final sample consisted of 330 participants, reflecting a completion rate of 59.6%.

### Measurements

All data were collected using a self-report questionnaire. The following assessments were used in the survey: eHL, constructs to examine convergent validity, constructs to examine criterion validity, and sociodemographic and COVID-19–related variables.

#### eHealth Literacy

The 8-item GR-eHEALS [23,49] was applied. This instrument reaches good psychometric properties regarding its validity and reliability as a 2-factor model covering the competences *information seeking* (IS) and *information appraisal* (IA) [49]. The response scale ranged from 1 for “strongly disagree” to 5 for “strongly agree,” with higher values corresponding to higher eHL.

#### Constructs to Examine Convergent Validity

We assessed the *knowledge of internet-based health promotion programs* via 3 self-developed items using a 5-point Likert scale (1 for “strongly disagree” to 5 for “strongly agree”). Higher scores indicated higher knowledge of internet-based health promotion programs. The items covered knowledge of the contents of such programs (“I can certainly imagine something under that.”), how they work (“I know how such programs work.”), and how to find them (“I know how to find such programs.”). Cronbach α was .88. The instrument has been previously used [50,51].

One self-developed item (“Have you already had experience with internet-based health promotion programs?”) was used to assess *experience in using internet-based health promotion programs* on a 3-point Likert scale (1 for “already used such programs,” 2 for “not used but aware of the possibilities of such programs,” and 3 for “not aware of the possibilities of such programs”). This item was inverted, so higher values represented more experience with internet-based health promotion programs. The assessment has been used in previous studies [50,51].

We obtained information about the *duration of daily private internet use* through a self-developed single item: “How long do you use the internet for private purposes every day?”. The response scale ranged from 1 for “not at all” to 5 for “more than 5 hours.” Previous studies have used this item [50,51].

The construct of *internet anxiety* was measured on a 5-point Likert scale (1 for “does not apply” to 5 for “applies”) using 3 self-developed items (“I have concerns about using the internet,” “I am afraid that I could make an irrevocable mistake when using the internet,” and “The internet is something that worries me.”). Higher scores represented higher internet anxiety. Cronbach α was .82. These items were applied previously [49-51].

We expected significant positive relationships between eHL and knowledge of internet-based health promotion programs, experience in using internet-based health promotion programs, and duration of daily private internet use. Further, a significant negative relationship between eHL and internet anxiety was expected.

#### Constructs to Examine Criterion Validity

We assessed the *internal health locus of control* with 3 items on a 5-point Likert scale (1 for “strongly disagree” to 5 for “strongly agree”) via an adapted German version of the Multidimensional Health Locus of Control Scale [52]. Higher scores reflected a higher internal health locus of control. Cronbach α was .76.

Moreover, we measured the *physical health status* (“On a scale of 0 to 10, how do you rate your physical health [eg, no physical limitations, pain]?”), *mental health status* (“On a scale of 0 to 10, how do you rate your mental health [eg, no feelings of anxiety, depression]?”), and *overall quality of life* (“On a scale of 0 to 10, how would you rate your current quality of life?”) with self-developed single items on 11-point Likert scales. These items have been used in previous studies [49,53].

We expected significant positive relationships between eHL and all criterion validity constructs.

#### Sociodemographic and COVID-19–Related Variables

We assessed participants’ main sociodemographic variables: age (as an exact number), gender (female, male, diverse), and marital status (married, living in a relationship, single, divorced/widowed, other). Further, participants’ educational degree (no school degree, secondary school certificate [*Hauptschule*], secondary school certificate [*Mittlere Reife*], university entrance qualification, university degree, academic degree, other, not specified), current employment status (attending school/study, employed [part-time, full-time], sick leave, retirement/pension, not employed/other), and population of the community they live in (big city, medium city, small city, rural village) were assessed.

Regarding COVID-19, we asked participants for the date of the positive test result of SARS-CoV-2 with a nasopharyngeal swab, as well as symptoms related to the post–COVID-19 condition (inclusion criteria). In addition, we gathered information about whether participants required treatment in a hospital (yes, no) and, in addition, intensive care due to their COVID-19 infection (yes, no). Further, we asked participants for a self-assessment of the severity of their COVID-19 symptoms (1 for “no symptoms” to 4 for “severe symptoms”) as well as their current physical capacity (1 for “still significantly limited” to 3 for “good”).

There was no “I don't know” or similar option available for any of the items. The instruments and items used are provided in Multimedia Appendix 2.

### Statistical Analysis

We conducted all statistical analyses using R (R Foundation for Statistical Computing) and RStudio (Posit) [54,55] extended with several packages. We report the distribution of sociodemographic as well as COVID-19–related characteristics of the study sample. Further, descriptive statistics were used to determine the item statistics of the GR-eHEALS items.

Next, we followed analytical steps to examine the validity and reliability of the GR-eHEALS according to the study aims: First, we conducted confirmatory factor analysis (CFA) to evaluate the factorial structure and construct validity. In the first model, we tested the 1-factorial structure of all 8 items loading on a single factor, as proposed by the authors of the original instrument [23]. In the second model, we considered the 2-factorial structure with the 2 intercorrelated factors IS and IA, as validated in the GR-eHEALS [49]. For model evaluation, we considered the recommendations of Hu and Bentler [56] and defined a good model fit when reaching a comparative fit index (CFI) and a Tucker–Lewis index (TLI) of about 0.95. Further, we determined that the root mean square error of approximation (RMSEA) should be <0.06, while the standardized root mean square residual (SRMR) should be ≤0.08. For model estimation, we used the robust version of the means-adjusted unweighted least squares (ULSM) estimator as the items were measured on ordinal-scaled levels [57]. We reported Cronbach α to evaluate the reliability of the scales.

Second, regarding aims 2 and 3, we conducted Spearman rank correlation analyses to examine convergent and criterion validity. All analyses considered a significance level of *P*=.05, and missing values were treated via listwise deletion.

Lastly, tests of measurement invariance were deployed to assess measurement equivalence of the instrument regarding age, gender, and educational level of participants (aim 4). Measurement invariance is a statistical requirement for the correct interpretation of group differences measured by an instrument. Otherwise, if an instrument is not measurement invariant, mean differences found between different groups differences should not be interpreted, as it may be that these differences arise because the instrument measures differently in different groups [58,59]. To allow the interpretation of mean differences, a scalar level of invariance should be achieved [57]. Therefore, we performed measurement invariance tests for the gradually more restrictive levels of configural invariance, metric invariance, and, finally, scalar invariance [60]. As there is no consensus regarding cutoff criteria for the evaluation of model fits within measurement invariance testing [61], we considered 2 common approaches: First, we compared the change in the CFI between the more restrictive model and the less restrictive model, which should be ≤0.01 [62]. Second, we set the configural level of invariance as the baseline model that should meet the criteria of good model fit, as reported earlier. Subsequently, we performed *χ*² difference tests to compare the model of metric invariance against the model of metric invariance and then the model of scalar invariance against the model of metric invariance [61]. Significant *χ*² difference tests would reflect a significant change in the model fit, implying substantial deterioration of the model.

## Results

### Study Sample Description

The study participants’ mean age was 36.6 (SD 12.9, min.=18, max.=70 years). An overview of the participants’ sociodemographic background is reported in Table 1. Most participants did not need hospital or intensive care treatment but experienced mild-to-severe symptoms due to COVID-19 infection. Most participants also reported limited physical capacities. Table 2 presents COVID-19–related information of the study participants. On average, participants reported to have experienced 2.7 (range 1-7) of the defined post–COVID-19 symptoms.

**Table 1:** Sociodemographic description of study participants (N=329^a^).

Sociodemographic characteristics		Participants, n (%)
**Gender**		
	Female	264 (80.0)
	Male	63 (19.1)
	Diverse	2 (0.6)
**Marital status**		
	Married	106 (32.1)
	Living in a relationship	107 (32.4)
	Single	100 (30.3)
	Divorced/widowed	11 (3.3)
	Other	5 (1.5)
**Educational degree^b^**		
	Secondary school diploma	77 (23.3)
	University entrance qualification	97 (29.4)
	University degree	148 (44.8)
	Other/not specified	7 (2.1)
**Employment status**		
	Attending school/study	81 (24.5)
	Employed (part-time, full-time)	118 (35.8)
	Sick leave	91 (27.6)
	Retirement/pension	5 (1.5)
	Not employed/other	34 (10.3)
**Community size**		
	Big city (>100,000 inhabitants)	142 (43.0)
	Medium city (>20,000 inhabitants)	79 (23.9)
	Small city (>5000 inhabitants)	52 (15.8)
	Rural village (<5000 inhabitants)	56 (17.0)

^a^Missing: n=1 (0.3%).

^b^Statements were summarized: secondary school certificate (*Hauptschule*) and secondary school certificate (*Mittlere Reife*) as secondary school diploma, and university degree and academic degree as university degree.

**Table 2:** Post–COVID-19–related medical data of study participants (N=330).

Post–COVID-19–related information		Participants, n (%)
**Required treatment in hospital**		
	Yes	18 (5.5)
	No	312 (94.5)
**Required intensive care**		
	Yes	7 (2.1)
	No	323 (97.9)
**Severity of COVID-19 symptoms**		
	No symptoms	16 (4.8)
	Mild symptoms	137 (41.5)
	Moderate symptoms	156 (47.3)
	Severe symptoms	21 (6.4)
**Current physical capacity**		
	Good	58 (17.6)
	Average	106 (32.1)
	Still significantly limited	166 (50.3)

### Psychometric Properties of the German Revised eHealth Literacy Scale

Table 3 shows the item statistics of the 8 GR-eHEALS items. The exact item wordings of the GR-eHEALS are presented in the original study [49].

**Table 3:** Descriptive item statistics of the GR-eHEALS^a^ items including means (SDs), skewness, kurtosis, and distribution of responses for each item (N=330).

Item	Mean (SD)	Skew	Kurtosis	Response distribution (%)				
				1 (strongly disagree)	2 (disagree)	3 (neutral)	4 (agree)	5 (strongly agree)
GR-eHEALS1	3.93 (0.91)	–0.97	0.82	2	8	12	52	26
GR-eHEALS2	4.05 (0.82)	–1.05	1.53	1	5	10	55	28
GR-eHEALS3	3.70 (0.97)	–0.60	–0.20	2	12	20	47	19
GR-eHEALS4	3.80 (0.93)	–0.70	–0.01	1	11	16	51	21
GR-eHEALS5	3.81 (0.84)	–0.75	0.62	1	7	19	56	17
GR-eHEALS6	4.03 (0.90)	–1.06	1.04	1	7	10	51	31
GR-eHEALS7	3.91 (1.01)	–1.00	0.69	3	7	15	45	30
GR-eHEALS8	3.80 (0.96)	–0.78	0.28	2	9	18	48	22

^a^GR-eHEALS: German Revised eHealth Literacy Scale.

All items had a median of 4 and showed slightly negative skewness, indicating that participants experienced high levels of eHL.

To examine the factorial structure of the GR-eHEALS, 2 rounds of CFA were performed. The results are presented in Table 4.

**Table 4:** Results of CFA^a^ of the GR-eHEALS^b^.

Model	*χ*² (*df*)	CFI^c^	TLI^d^	RMSEA^e^	SRMR^f^
1-factor model^g^	218.14 (20)	0.98	0.97	0.088	0.079
2-factor model^h^	59.96 (19)	1.00	0.99	0.036	0.038

^a^CFA: confirmatory factor analysis.

^b^GR-eHEALS: German Revised eHealth Literacy Scale.

^c^CFI: comparative fit index.

^d^TLI: Tucker–Lewis index.

^e^RMSEA: root mean square error of approximation.

^f^SRMR: standardized root mean square residual.

^g^Model with all 8 items loading on 1 common factor.

^h^Model with 2 subscales, “information seeking” (items 1-4) and “information appraisal” (items 5-8).

The 2-factor model revealed an excellent model fit, whereas the 1-factor model slightly did not meet the recommendations regarding the RMSEA and SRMR. In addition, the *χ*² difference test, which compared the 2 models based on the nonrobust estimator, confirmed significant model improvement for the 2-factor model over the 1-factor model (*χ*²_1_=33.18, *P*<.001). Consequently, the 2-factor model with the 2 interrelated factors IS and IA was used for subsequent analyses. All items reached high factor loadings (≥.73) and the 2 factors were significantly correlated, with *r*=0.74 (*P*<.001). The item factor loadings are presented in Multimedia Appendix 3. IS (mean 3.87, SD 0.80) and IA (mean 3.89, SD 0.78) reached high reliability (Cronbach α=.90 and .86, respectively).

Due to the high correlation of the subscales, the overall GR-eHEALS with all 8 items was also reported for the investigation of convergent and criterion validity. The overall GR-eHEALS (mean 3.88, SD 0.72) achieved an excellent reliability of 0.91. Therefore, the construct validity and reliability of the GR-eHEALS was confirmed.

### Convergent and Criterion Validity of the GR-eHEALS in Individuals With the Post–COVID-19 Condition

To evaluate the convergent validity and criterion validity of the GR-eHEALS, Spearman rank correlation analyses were performed. The results are presented in Table 5.

**Table 5:** Spearman rank correlations of the GR-eHEALS^a^ subscales and the overall GR-eHEALS with convergent and criterion validity variables.

Validity and variables		IS^b^ subscale		IA^c^ subscale		Overall scale	
		Correlation coefficient, *r*	*P* value	Correlation coefficient, *r*	*P* value	Correlation coefficient, *r*	*P* value
**Convergent validity**							
	Knowledge of internet-based health promotion programs	0.45	<.001	0.31	<.001	0.41	<.001
	Experience in using internet-based health promotion programs	0.28	<.001	0.17	<.01	0.25	<.001
	Duration of daily private internet use	0.15	<.01	0.16	<.01	0.16	<.01
	Internet anxiety	–0.20	<.001	–0.30	<.001	–0.28	<.001
**Criterion validity**							
	Physical health	0.04	.47	–0.01	.86	0.02	.71
	Mental health	0.14	<.05	0.11	<.05	0.14	<.05
	Quality of life	0.12	<.05	0.06	.29	0.10	.06
	Internal health locus of control	0.19	<.001	0.18	<.001	0.20	<.001

^a^GR-eHEALS: German Revised eHealth Literacy Scale.

^b^IS: information seeking.

^c^IA: information appraisal.

IS and IA were both significantly positively associated with knowledge of and experience in using internet-based health promotion programs as well as with the duration of daily private internet use. Moreover, both factors were significantly negatively related to internet anxiety. The results were supported by similar relationships regarding the overall GR-eHEALS. These results confirm the convergent validity of the GR-eHEALS regarding the study aim 2.

Although criterion validity was supported by significantly positively relations of the IS and IA with mental health and the internal health locus of control, quality of life was only related to IS, and physical health was not related to any of the 2 factors. The overall GR-eHEALS was similarly significantly related to mental health status and the internal health locus of control but not to physical health and quality of life. Consequently, results regarding aim 3 were inconsistent.

To further understand these results, we conducted correlation analyses between all criterion validity scales. The results revealed that lower physical health is strongly associated with lower mental health (*r*=0.40, *P*<.001), a lower quality of life (*r*=0.69, *P*<.001), and a lower internal health locus of control (*r*=0.31, *P*<.001). The correlation table is presented in Multimedia Appendix 4.

IS and IA were both not associated with any of the post–COVID-19–related characteristics (need for intensive care was not tested due to the small subgroup sample size).

### Measurement Invariance of the GR-eHEALS in Individuals With the Post–COVID-19 Condition

For testing measurement invariance regarding gender, we excluded participants who indicated their gender as diverse in order to have sufficient group sample sizes for this analysis. Regarding age, we performed a median split to divide the sample at the median into 2 groups (median age 34 years). Regarding educational level, we divided the sample into 2 groups: group 1 consisted of participants with any school certificate, and group 2 consisted of participants holding a university degree. Participants who indicated other educational levels than these were excluded from this analysis. Multimedia Appendix 5 summarizes the results of the tests of measurement invariance of the GR-eHEALS regarding gender, age, and educational level.

All changes in the CFI were below 0.01, indicating measurement invariance for gender, age, and educational level. Nevertheless, *χ*² difference tests showed that the configural level of invariance for all 3 sociodemographic variables reached good model fit indices, implying that this level of invariance could be confirmed regarding all 3 sociodemographic variables. Regarding gender, subsequent *χ*² difference tests were not significant for metric and scalar levels of invariance, which is evidence for the assumption of measurement invariance of the GR-eHEALS. With respect to age and educational level, the *χ*² difference tests were not significant for the metric levels of measurement invariance but were significant for the scalar levels. This indicated that only partial measurement invariance of the GR-eHEALS regarding age and educational level could be confirmed. Hence, results regarding aim 4 were inconsistent.

## Discussion

### Principal Findings

This is the first study to evaluate the validity and reliability of the internationally most common instrument for measuring eHL in individuals with the post–COVID-19 condition. Thus, there is a gap in the knowledge of whether this instrument is still appropriate in measuring eHL in an era of an increasing number of individuals with the post–COVID-19 condition. Our study addresses this research gap and provides the first evidence by examining the measurement properties of the German version of eHEALS in individuals with the post–COVID-19 condition. The CFA results underpin the assumed factorial structure of the GR-eHEALS [49], supporting the construct validity of the instrument (aim 1). The reliability of the GR-eHEALS could be confirmed with high coefficients for both factors, IS and IA, as well as for the overall scale.

The subsequently performed correlation analyses with 4 convergent validity scales showed highly consistent results and confirmed the convergent validity of the GR-eHEALS (aim 2).

Concerning the criterion validity of the GR-eHEALS (aim 3), results did not fully meet our assumptions: physical health was not related to the GR-eHEALS, and the overall quality of life was only related to IS. However, our results are not the first that showed no relation between eHL and the physical health status [35]. In addition, this result could be explained by the fact that the study sample consisted of individuals with an impaired physical health status due to the inclusion criteria. Many of the study participants still experienced limitations in their physical capacity at the time of completing the questionnaire. Further, as shown in Multimedia Appendix 4, quality of life and physical health were strongly interrelated. That could explain why quality of life also did not confirm the expected relation to IA. In summary, most individuals in the study sample still had symptoms of the post–COVID-19 condition, which may not be affected by their eHL. It is possible that people who currently have diseases with physical health limitations cannot compensate for those limitations by high levels of eHL. Nevertheless, we found significant relations between the GR-eHEALS and mental health as well as the internal health locus of control. A high internal health locus of control reflects individuals’ confidence to influence their own health, which is associated with higher mental health status [63], better health behaviors [64], higher levels of physical activity [65], lower perceived stress [66], and higher medication adherence [67]. The fact that the internal health locus of control was strongly associated with eHL implies that this competence may play a central role when individuals are facing ambiguous information from the internet and still experience themselves as competent in handling that information and their health.

Regarding aim 4, the results of the measurement invariance tests of the GR-eHEALS were partly confirmed. Measurement invariance is important to assume that assessments measure the same underlying constructs consistently over different groups and populations [68]. If an assessment is not measurement invariant, differences between groups may be due to a bias in the assessment tool rather than true differences in the construct, which could lead to inaccurate interpretation of study results and potentially misleading recommendations for public health [69]. Measurement invariance is also an important prerequisite for the generalizability of study results [70]. This is particularly important for public health research, since study results are used to inform public policies and for the development of interventions, such as for health promotion [71,72]. Our results indicate that there are no substantial differences in the measurement model of the GR-eHEALS regarding gender. However, regarding age and educational level, measurement invariance was only partly confirmed but missed achieving scalar invariance. These results are important as studies have revealed that COVID-19 infection and its consequences are determined by gender, age, and educational level [73-77]. With the confirmation of measurement invariance of the GR-eHEALS regarding gender, we provide a valid instrument to assess and interpret differences in gender. With regard to age and educational level, group differences may only be interpreted under consideration of the partly confirmed invariance.

Summarizing, our results underpin the general construct validity, convergent validity, and reliability of the GR-eHEALS in individuals with the post–COVID-19 condition. The criterion validity of the instrument could mainly be confirmed. By partly confirming the measurement invariance, we showed that the GR-eHEALS is a valid instrument to assess and interpret eHL but is limited in terms of the interpretation of differences in age and educational level.

The overall GR-eHEALS showed consistent results in terms of convergent and criterion validity. Provided the focus is on a general examination of eHL, the overall scale can therefore be used appropriately. Nevertheless, depending on the research question, the subscales offer an opportunity to consider the competence domains of eHL in a differentiated manner.

The mean values and factor loadings of the items as well as the interrelation of the GR-eHEALS subscales were similar of those reported in the initial validation of the GR-eHEALS [49]. This is an indication that the GR-eHEALS performs and can be used similarly across different studies and in different populations. Nevertheless, the measurement invariance of the instrument across different populations should be investigated in future studies before comparing the results of different study groups. However, as the factorial structure of eHEALS is not consistent in different languages [23,78,79], further studies are needed to verify the validity of the instrument in the population with the post–COVID-19 condition.

It is important to note that the GR-eHEALS is based on the original eHEALS, which was published back in 2006 [23]. Now, the (digital) world has changed significantly. New information sources have emerged, and social media platforms have become a considerable source for health information gathering [80]. This is particularly relevant because the COVID-19 pandemic has contributed to the fact that people no longer simply obtain health information from the Internet but increasingly use social media (eg, Instagram, YouTube, Twitter) and follow individual influencers to acquire health information [81]. Therefore, the appropriateness of the use of eHEALS should be considered critically and subject to future research. Newer instruments for measuring eHL have appeared claiming to assess this construct more comprehensively and adapt to new environmental conditions [45]. Future studies should therefore establish comparative analyses of the psychometric properties of different instruments, especially in consideration of the social as well as digital environments individuals are facing in the postpandemic world. However, the study results indicate largely the validity and reliability of the GR-eHEALS to assess eHL in people after COVID-19 infection. As eHEALS is still the most widely used instrument worldwide to assess eHL [44], it offers the possibility of comparing results between different countries and populations. eHEALS thus remains an important instrument for the assessment of eHL.

### Limitations

The study sample covers a wide range of sociodemographic characteristics. Nevertheless, female participants were overrepresented in the sample, and most participants were middle aged, resulting in a small number of individuals who were already retired. This could represent a bias as young people interact with digital media differently than older people [82]. In addition, a high proportion of individuals with a university degree was present in the sample. Our survey was only accessible online, so the sample may be biased toward individuals with higher affinity to the use of digital media. Moreover, responses to the survey may have been biased by participants’ technology skills. These circumstances may limit the generalizability of our results. Exclusively self-report instruments were used, which may have introduced a response bias. As we also recorded via self-report whether a confirmed COVID-19 infection and post–COVID-19 symptoms were present, the inclusion criteria for this study were particularly affected by this restriction. Moreover, this cross-sectional study does not allow for the interpretation of causal relationships. Therefore, all interrelations found only reflect relationships without implying causal directions. The validation methods applied were based on the assumptions of classical test theory, and future studies should consider alternative paradigms, such as item response theory. Further, even if already used in other studies, the instruments used to test convergent and criterion validity were mostly not validated instruments. This could limit the external validity of the results.

### Conclusion

The validity and reliability of the GR-eHEALS could mainly be confirmed in a sample of individuals with the post–COVID-19 condition. This is the first study to examine eHL in this population, which is particularly vulnerable to misinformation. By partly confirming the measurement invariance of the instrument, we provided evidence that the GR-eHEALS is an important instrument in public health research due to its ability to be interpretable regarding differences in gender and partly regarding age and educational level. However, future research should also explore alternative instruments to capture eHL and thus consider changing behaviors of disseminating and obtaining digital health information, for example, through social media.

## Appendix

Multimedia Appendix 1 Number of participants reporting each post–COVID-19 symptom.

Multimedia Appendix 2 Study survey.

Multimedia Appendix 3 Item factor loadings of the German Revised eHealth Literacy Scale (GR-eHEALS).

Multimedia Appendix 4 Correlation table of criterion validity scales.

Multimedia Appendix 5 Results of tests of measurement invariance of the German Revised eHealth Literacy Scale (GR-eHEALS) regarding gender, age, and educational level.

## Abbreviations

CFA: confirmatory factor analysis
CFI: comparative fit index
eHL: eHealth Literacy
eHEALS: eHealth Literacy Scale
GR-eHEALS: German Revised eHealth Literacy Scale
IA: information appraisal
IS: information seeking
RMSEA: root mean square error of approximation
SRMR: standardized root mean square residual
TLI: Tucker–Lewis index
ULSM: unweighted least squares estimator

## Data Availability

The data sets used and analyzed during this current study are available from the corresponding author upon reasonable request.

## References

[1] Pavli A, Theodoridou M, Maltezou HC (2021). Post-COVID syndrome: incidence, clinical spectrum, and challenges for primary healthcare professionals. Arch Med Res.

[2] Soriano JB, Murthy S, Marshall JC, Relan P, Diaz JV, WHO Clinical Case Definition Working Group on Post-COVID-19 Condition (2022). A clinical case definition of post-COVID-19 condition by a Delphi consensus. Lancet Infect Dis.

[3] Nalbandian A, Sehgal K, Gupta A, Madhavan MV, McGroder C, Stevens JS, Cook JR, Nordvig AS, Shalev D, Sehrawat TS, Ahluwalia N, Bikdeli B, Dietz D, Der-Nigoghossian C, Liyanage-Don N, Rosner GF, Bernstein EJ, Mohan S, Beckley AA, Seres DS, Choueiri TK, Uriel N, Ausiello JC, Accili D, Freedberg DE, Baldwin M, Schwartz A, Brodie D, Garcia CK, Elkind MSV, Connors JM, Bilezikian JP, Landry DW, Wan EY (2021). Post-acute COVID-19 syndrome. Nat Med.

[4] TK-Gesundheitsreport 2022: Krankenstand durch Long-COVID. Die Techniker - Presse & Politik.

[5] Westerlind E, Palstam A, Sunnerhagen KS, Persson HC (2021). Patterns and predictors of sick leave after Covid-19 and long Covid in a national Swedish cohort. BMC Public Health.

[6] Gaber T, Ashish A, Unsworth A (2021). Persistent post-covid symptoms in healthcare workers. Occup Med (Lond).

[7] Matta J, Wiernik E, Robineau O, Carrat F, Touvier M, Severi G, de Lamballerie X, Blanché H, Deleuze J, Gouraud C, Hoertel N, Ranque B, Goldberg M, Zins M, Lemogne C, Santé‚ Pratiques‚ Relations et Inégalités Sociales en Population Générale Pendant la Crise COVID-19–Sérologie (SAPRIS-SERO) Study Group (2022). Association of self-reported COVID-19 infection and SARS-CoV-2 serology test results with persistent physical symptoms among French adults during the COVID-19 pandemic. JAMA Intern Med.

[8] Staudt A, Jörres RA, Hinterberger T, Lehnen N, Loew T, Budweiser S (2022). Associations of post-acute COVID syndrome with physiological and clinical measures 10 months after hospitalization in patients of the first wave. Eur J Intern Med.

[9] Cinelli M, Quattrociocchi W, Galeazzi A, Valensise CM, Brugnoli E, Schmidt AL, Zola P, Zollo F, Scala A (2020). The COVID-19 social media infodemic. Sci Rep.

[10] Zhang S, Pian W, Ma F, Ni Z, Liu Y (2021). Characterizing the COVID-19 infodemic on Chinese social media: exploratory study. JMIR Public Health Surveill.

[11] van der Linden S, Roozenbeek J, Compton J (2020). Inoculating against fake news about COVID-19. Front Psychol.

[12] Charquero-Ballester M, Walter JG, Nissen IA, Bechmann A (2021). Different types of COVID-19 misinformation have different emotional valence on Twitter. Big Data Soc.

[13] Rovetta A, Bhagavathula AS (2020). Global infodemiology of COVID-19: analysis of Google web searches and Instagram hashtags. J Med Internet Res.

[14] Rathore FA, Farooq F (2020). Information overload and infodemic in the COVID-19 pandemic. J Pak Med Assoc.

[15] The Lancet Infectious Diseases (2020). The COVID-19 infodemic. Lancet Infect Dis.

[16] Eysenbach G (2020). How to fight an infodemic: the four pillars of infodemic management. J Med Internet Res.

[17] Islam MS, Sarkar T, Khan SH, Mostofa Kamal A, Hasan SMM, Kabir A, Yeasmin D, Islam MA, Amin Chowdhury KI, Anwar KS, Chughtai AA, Seale H (2020). COVID-19-related infodemic and its impact on public health: a global social media analysis. Am J Trop Med Hyg.

[18] Mheidly N, Fares J (2020). Leveraging media and health communication strategies to overcome the COVID-19 infodemic. J Public Health Policy.

[19] Wang Y, McKee M, Torbica A, Stuckler D (2019). Systematic literature review on the spread of health-related misinformation on social media. Soc Sci Med.

[20] Norman CD, Skinner HA (2006). eHealth literacy: essential skills for consumer health in a networked world. J Med Internet Res.

[21] Mitsutake S, Shibata A, Ishii K, Oka K (2016). Associations of eHealth literacy with health behavior among adult internet users. J Med Internet Res.

[22] Kim K, Shin S, Kim S, Lee E (2023). The relation between eHealth literacy and health-related behaviors: systematic review and meta-analysis. J Med Internet Res.

[23] Norman CD, Skinner HA (2006). eHEALS: the eHealth Literacy Scale. J Med Internet Res.

[24] Rathnayake S, Liyanage IP (2022). Cross-cultural adaptation and psychometric properties of the Sinhala version of electronic health literacy scale: a cross-sectional validation study. PLoS One.

[25] Shiferaw KB (2020). Validation of the Ethiopian version of eHealth Literacy Scale (ET-eHEALS) in a population with chronic disease. Risk Manag Healthc Policy.

[26] Wangdahl JM, Dahlberg K, Jaensson M, Nilsson U (2019). Psychometric validation of Swedish and Arabic versions of two health literacy questionnaires, eHEALS and HLS-EU-Q16, for use in a Swedish context: a study protocol. BMJ Open.

[27] Del Giudice P, Bravo G, Poletto M, De Odorico A, Conte A, Brunelli L, Arnoldo L, Brusaferro S (2018). Correlation between eHealth literacy and health literacy using the eHealth Literacy Scale and real-life experiences in the health sector as a proxy measure of functional health literacy: cross-sectional web-based survey. J Med Internet Res.

[28] Paramio Pérez G, Almagro BJ, Hernando Gómez Á, Aguaded Gómez JI (2015). [Validation of the eHealth Literacy Scale (eHEALS) in Spanish university students]. Rev Esp Salud Publica.

[29] van der Vaart R, van Deursen AJ, Drossaert CH, Taal E, van Dijk JA, van de Laar MA (2011). Does the eHealth Literacy Scale (eHEALS) measure what it intends to measure? Validation of a Dutch version of the eHEALS in two adult populations. J Med Internet Res.

[30] Chang A, Schulz PJ (2018). The measurements and an elaborated understanding of Chinese eHealth literacy (C-eHEALS) in chronic patients in China. Int J Environ Res Public Health.

[31] Gazibara T, Cakic J, Cakic M, Pekmezovic T, Grgurevic A (2019). eHealth and adolescents in Serbia: psychometric properties of eHeals questionnaire and contributing factors to better online health literacy. Health Promot Int.

[32] Chung S, Park BK, Nahm E (2018). The Korean eHealth Literacy Scale (K-eHEALS): reliability and validity testing in younger adults recruited online. J Med Internet Res.

[33] Wijaya MC, Kloping YP (2021). Validity and reliability testing of the Indonesian version of the eHealth Literacy Scale during the COVID-19 pandemic. Health Inform J.

[34] Cui G, Li S, Yin Y, Chen L, Li J, Liang F, Liu X, Chen L (2021). The relationship among social capital, eHealth literacy and health behaviours in Chinese elderly people: a cross-sectional study. BMC Public Health.

[35] Xie L, Zhang S, Xin M, Zhu M, Lu W, Mo PK (2022). Electronic health literacy and health-related outcomes among older adults: a systematic review. Prev Med.

[36] Tsukahara S, Yamaguchi S, Igarashi F, Uruma R, Ikuina N, Iwakura K, Koizumi K, Sato Y (2020). Association of eHealth literacy with lifestyle behaviors in university students: questionnaire-based cross-sectional study. J Med Internet Res.

[37] Brørs G, Wentzel-Larsen T, Dalen H, Hansen TB, Norman CD, Wahl A, Norekvål TM, CONCARD Investigators (2020). Psychometric properties of the Norwegian version of the electronic Health Literacy Scale (eHEALS) among patients after percutaneous coronary intervention: cross-sectional validation study. J Med Internet Res.

[38] Guo Z, Zhao SZ, Guo N, Wu Y, Weng X, Wong JY, Lam TH, Wang MP (2021). Socioeconomic disparities in eHealth literacy and preventive behaviors during the COVID-19 pandemic in Hong Kong: cross-sectional study. J Med Internet Res.

[39] Li S, Cui G, Kaminga AC, Cheng S, Xu H (2021). Associations between health literacy, eHealth literacy, and COVID-19–related health behaviors among Chinese college students: cross-sectional online study. J Med Internet Res.

[40] Moghadas S, Vilches T, Zhang K, Wells C, Shoukat A, Singer B, Meyers L, Neuzil K, Langley J, Fitzpatrick M, Galvani A (2021). The impact of vaccination on coronavirus disease 2019 (COVID-19) outbreaks in the United States. Clin Infect Dis.

[41] Nath R, Imtiaz A, Nath SD, Hasan E (2021). Role of vaccine hesitancy, eHealth literacy, and vaccine literacy in young adults’ COVID-19 vaccine uptake intention in a lower-middle-income country. Vaccines (Basel).

[42] Rosen AO, Holmes AL, Balluerka N, Hidalgo MD, Gorostiaga A, Gómez-Benito J, Huedo-Medina TB (2022). Is social media a new type of social support? Social media use in Spain during the COVID-19 pandemic: a mixed methods study. Int J Environ Res Public Health.

[43] Van Aelst P, Toth F, Castro L, Štětka V, Vreese CD, Aalberg T, Cardenal AS, Corbu N, Esser F, Hopmann DN, Koc-Michalska K, Matthes J, Schemer C, Sheafer T, Splendore S, Stanyer J, Stępińska A, Strömbäck J, Theocharis Y (2021). Does a a crisis change news habits? A comparative study of the effects of COVID-19 on news media use in 17 European countries. Digital Journalism.

[44] Lee J, Lee E, Chae D (2021). eHealth literacy instruments: systematic review of measurement properties. J Med Internet Res.

[45] Paige SR, Stellefson M, Krieger JL, Miller MD, Cheong J, Anderson-Lewis C (2019). Transactional eHealth literacy: developing and testing a multi-dimensional instrument. J Health Commun.

[46] Karnoe A, Kayser L (2015). How is eHealth literacy measured and what do the measurements tell us? A systematic review. Knowl Manag E-Learn Int J.

[47] Neter E, Brainin E (2019). Association between health literacy, eHealth literacy, and health outcomes among patients with long-term conditions: a systematic review. Eur Psychol.

[48] Tariq A, Khan SR, Basharat A (2020). Internet use, eHealth literacy, and dietary supplement use among young adults in Pakistan: cross-sectional study. J Med Internet Res.

[49] Marsall M, Engelmann G, Skoda E, Teufel M, Bäuerle A (2022). Measuring electronic health literacy: development, validation, and test of measurement invariance of a revised German version of the eHealth Literacy Scale. J Med Internet Res.

[50] Rentrop V, Damerau M, Schweda A, Steinbach J, Schüren LC, Niedergethmann M, Skoda E, Teufel M, Bäuerle A (2022). Predicting acceptance of e–mental health interventions in patients with obesity by using an extended unified theory of acceptance model: cross-sectional study. JMIR Form Res.

[51] Bäuerle A, Frewer A, Rentrop V, Schüren LC, Niedergethmann M, Lortz J, Skoda E, Teufel M (2022). Determinants of acceptance of weight management applications in overweight and obese individuals: using an extended unified theory of acceptance and use of technology model. Nutrients.

[52] Hasenbring M, Schüffel W (1988). Zur Adaptivität von Kontrollüberzeugungen — Empirische Befunde bei Patienten mit Krebserkrankungen, lumbalem Bandscheibenvorfall und chronischen Schmerzsyndromen. Sich Gesund Fühlen Im Jahre 2000.

[53] Engelmann G, Marsall M, Skoda E, Knoll-Pientka N, Bäuerle L, Stroebele-Benschop N, Teufel M, Bäuerle A (2021). Development and validation of the General Dietary Behavior Inventory (GDBI) in scope of international nutrition guidelines. Nutrients.

[54] R: a language and environment for statistical computing. R Core Team.

[55] RSTUDIO IDE: the most trusted IDE for open source data science. Posit.

[56] Hu L, Bentler PM (1999). Cutoff criteria for fit indexes in covariance structure analysis: conventional criteria versus new alternatives. Struct Equ Model.

[57] Sellbom M, Tellegen A (2019). Factor analysis in psychological assessment research: common pitfalls and recommendations. Psychol Assess.

[58] Van De Schoot R, Schmidt P, De Beuckelaer A, Lek K, Zondervan-Zwijnenburg M (2015). Editorial: measurement invariance. Front Psychol.

[59] Hirschfeld G, Von BR (2014). Improving multiple-group confirmatory factor analysis in R–a tutorial in measurement invariance with continuous and ordinal indicators. Pract Assess Res Eval.

[60] Dimitrov DM (2017). Testing for factorial invariance in the context of construct validation. Meas Eval Counsel Dev.

[61] Svetina D, Rutkowski L, Rutkowski D (2019). Multiple-group invariance with categorical outcomes using updated guidelines: an illustration using M plus and the lavaan/semTools packages. Struct Equ Model.

[62] Putnick DL, Bornstein MH (2016). Measurement invariance conventions and reporting: the state of the art and future directions for psychological research. Dev Rev.

[63] Wilski M, Brola W, Tomczak M (2019). Health locus of control and mental health in patients with multiple sclerosis: mediating effect of coping strategies. Res Nurs Health.

[64] Milaniak I, Dębska G, Król B, Wierzbicki K, Przybyłowski P (2022). Health locus of control and health behaviors in organ transplant recipients: a multicenter study. Transplant Proc.

[65] Mercer DA, Ditto B, Lavoie KL, Campbell T, Arsenault A, Bacon SL (2018). Health locus of control is associated with physical activity and other health behaviors in cardiac patients. J Cardiopulm Rehabil Prev.

[66] Ganjoo M, Farhadi A, Baghbani R, Daneshi S, Nemati R (2021). Association between health locus of control and perceived stress in college student during the COVID-19 outbreak: a cross-sectional study in Iran. BMC Psychiatry.

[67] Náfrádi L, Nakamoto K, Schulz PJ (2017). Is patient empowerment the key to promote adherence? A systematic review of the relationship between self-efficacy, health locus of control and medication adherence. PLoS One.

[68] Milfont TL, Fischer R (2010). Testing measurement invariance across groups: applications in cross-cultural research. Int J Psychol Res.

[69] Davidov E, Meuleman B, Cieciuch J, Schmidt P, Billiet J (2014). Measurement equivalence in cross-national research. Annu Rev Sociol.

[70] Vandenberg RJ, Lance CE (2016). A review and synthesis of the measurement invariance literature: suggestions, practices, and recommendations for organizational research. Org Res Methods.

[71] Dobrow MJ, Miller FA, Frank C, Brown AD (2017). Understanding relevance of health research: considerations in the context of research impact assessment. Health Res Policy Syst.

[72] Weise A, Büchter R, Pieper D, Mathes T (2020). Assessing context suitability (generalizability, external validity, applicability or transferability) of findings in evidence syntheses in healthcare—an integrative review of methodological guidance. Res Synth Methods.

[73] Khanijahani A, Iezadi S, Gholipour K, Azami-Aghdash S, Naghibi D (2021). A systematic review of racial/ethnic and socioeconomic disparities in COVID-19. Int J Equity Health.

[74] De Negri F, Galiezz R, Miranda P, Koeller P, Zucoloto G, Costa J, Farias CM, Travassos GH, Medronho RA (2021). Socioeconomic factors and the probability of death by COVID-19 in Brazil. J Public Health (Oxf).

[75] Hawkins RB, Charles EJ, Mehaffey JH (2020). Socio-economic status and COVID-19-related cases and fatalities. Public Health.

[76] Jin J, Bai P, He W, Wu F, Liu X, Han D, Liu S, Yang J (2020). Gender differences in patients with COVID-19: focus on severity and mortality. Front Public Health.

[77] Sykes DL, Holdsworth L, Jawad N, Gunasekera P, Morice AH, Crooks MG (2021). Post-COVID-19 symptom burden: what is long-COVID and how should we manage it?. Lung.

[78] Ma Z, Wu M (2019). The psychometric properties of the Chinese eHealth Literacy Scale (C-eHEALS) in a Chinese rural population: cross-sectional validation study. J Med Internet Res.

[79] Dale JG, Lüthi A, Fundingsland Skaraas B, Rundereim T, Dale B (2020). Testing measurement properties of the Norwegian version of electronic Health Literacy Scale (eHEALS) in a group of day surgery patients. J Multidiscip Healthc.

[80] Niu Z, Willoughby J, Zhou R (2021). Associations of health literacy, social media use, and self-efficacy with health information–seeking intentions among social media users in China: cross-sectional survey. J Med Internet Res.

[81] Lim MSC, Molenaar A, Brennan L, Reid M, McCaffrey T (2022). Young adults’ use of different social media platforms for health information: insights from web-based conversations. J Med Internet Res.

[82] Yates S, Kirby J, Lockley E (2015). Digital media use: differences and inequalities in relation to class and age. Sociol Res Online.

