# Supplementary material for: Assessing Electronic Health Literacy in Individuals With the Post–COVID-19 Condition Using the German Revised eHealth Literacy Scale: Validation Study
Source: JMIR Form Res. 2024 Apr 25;8:e52189. doi: 10.2196/52189 (PMC11082733; doi:10.2196/52189)
Supplement: Multimedia Appendix 1 [file formative_v8i1e52189_app1.docx]

| **Post COVID-19 symptoms** | **N (%)** |
| --- | --- |
| Sore throat | 93 (28.2) |
| Cough | 114 (34.5) |
| Shortness of breath | 114 (34.5) |
| Headache / Pain in the Limbs | 191 (57.9) |
| Body temperature above 38 °C | 33 (10.0) |
| Olfactory and/or gustatory disturbances | 109 (33.0) |
| Diarrhea | 43 (13.0) |
| Other | 187 (56.7) |
