# Supplementary material for: Assessing Electronic Health Literacy in Individuals With the Post–COVID-19 Condition Using the German Revised eHealth Literacy Scale: Validation Study
Source: JMIR Form Res. 2024 Apr 25;8:e52189. doi: 10.2196/52189 (PMC11082733; doi:10.2196/52189)
Supplement: Multimedia Appendix 2 [file formative_v8i1e52189_app2.docx]

1. **eHealth Literacy**

Please read through the individual statements and mark how much you agree with each statement.

| **#** | **German Items (GR-eHEALS)**^1^ | **English Items of eHEALS**^2^ | **Response** |
| --- | --- | --- | --- |
| 1 | Ich weiß, wie ich Internetseiten mit Hilfreichen Gesundheitsinformationen finden kann. | I know how to find helpful health resources on the Internet. | 1 = „Strongly disagree“,  2 = „Disagree“,  3 = „Neutral“,  4 = „Agree“,  5 = „Strongly agree“ |
| 2 | Ich weiß, wie ich das Internet nutzen kann, um Antworten auf meine Gesundheitsfragen zu erhalten. | I know how to use the Internet to answer my health questions. |  |
| 3 | Ich weiß, welche Seiten mit Gesundheitsinformationen im Internet verfügbar sind. | I know what health resources are available on the Internet. |  |
| 4 | Ich weiß, wo ich im Internet hilfreiche Gesundheitsinformationen finden kann. | I know where to find helpful health resources on the Internet. |  |
| 5 | Ich weiß, Gesundheitsinformationen aus dem Internet so zu nutzen, dass sie mir weiterhelfen. | I know how to use the health information I find on the Internet to help me. |  |
| 6 | Ich bin in der Lage, Internetseiten mit Gesundheitsinformationen kritisch zu bewerten. | I have the skills I need to evaluate the health resources I find on the Internet. |  |
| 7 | Ich kann zwischen vertrauenswürdigen und fragwürdigen Internetseiten mit Gesundheitsinformationen unterscheiden. | I can tell high quality from low quality health resources on the Internet. |  |
| 8 | Ich fühle mich sicher darin, Informationen aus dem Internet zu nutzen, um Entscheidungen in Bezug auf meine Gesundheit zu treffen. | I feel confident in using information from the Internet to make health decisions. |  |

^1^ Marsall, Engelmann, Skoda, Teufel, and Bäuerle. „Measuring Electronic Health Literacy: Development, Validation, and Test of Measurement Invariance of a Revised German Version of the eHealth Literacy Scale“. Journal of Medical Internet Research https://doi.org/10.2196/28252.);

^2^ (Norman & Skinner. „eHEALS: The eHealth Literacy Scale“. Journal of Medical Internet Research https://doi.org/10.2196/jmir.8.4.e27)

1. **Knowledge of internet-based health promotion programs**

What do you already know about internet-based health promotion programs?

| **#** | **Translated Items** | **Response** |
| --- | --- | --- |
| 1 | I can certainly imagine something under that. | 1 = „Strongly disagree“,  2 = „Disagree“,  3 = „Neutral“,  4 = „Agree“,  5 = „Strongly agree“ |
| 2 | I know how such programs work. |  |
| 3 | I know how to find such programs. |  |

1. **Experience in using internet-based health promotion programs**

| **#** | **Translated Item** | **Response** |
| --- | --- | --- |
| 1 | Have you already had experience with Internet-based health promotion programs? | 1 = “Already used such programs”,  2 = “Not used, but aware of the possibilities of such programs”,  3 = “Not aware on the possibilities of such programs” |

1. **Duration of daily private internet use**

| **#** | **Translated Item** | **Response** |
| --- | --- | --- |
| 1 | How long do you use the Internet for private purposes every day? | 1 = „Not at all“,  2 = „Less than 1 hour“,  3 = „1 to 3 hours“,  4 = „3 to 5 hours“,  5 = „More than 5 hours“ |

1. **Internet anxiety**

To what extent do each of the following statements apply to you?

| **#** | **Translated Items** | **Response** |
| --- | --- | --- |
| 1 | I have concerns about using the Internet. | 1 = „Does not apply“,  2 = „Rather does not apply“,  3 = „Neutral“,  4 = „Rather applies“,  5 = „Applies“ |
| 2 | I am afraid that I could make an irrevocable mistake when using the Internet. |  |
| 3 | The Internet is something that worries me. |  |

1. **Internal health locus of control**

Some attitudes towards health and illness are described below. Some statements are very general, others refer to your current illness. Please read through each sentence and assess how much the statement applies to you at the moment.

| **#** | **Translated Item of the adapted German version of the Multidimensional Health Locus of Control Scale^1^** | **Response** |
| --- | --- | --- |
| 1 | I can do a lot myself to influence my well-being. | 1 = „Strongly disagree“,  2 = „Disagree“,  3 = „Neutral“,  4 = „Agree“,  5 = „Strongly agree“ |
| 2 | I myself have a big influence on how my illness will progress. |  |
| 3 | If I take care of myself, I can avoid pain. |  |

^1^ Hasenbring M. Zur Adaptivität von Kontrollüberzeugungen — Empirische Befunde bei Patienten mit Krebserkrankungen, lumbalem Bandscheibenvorfall und chronischen Schmerzsyndromen. In: Schüffel W, editor. Sich Gesund Fühlen Im Jahre 2000 Berlin, Heidelberg: Springer; 1988. p. 222–230

1. **Health status**

| **#** | **Translated Items** | **Response** |
| --- | --- | --- |
| 1 | On a scale of 0 to 10, how do you rate your physical health (eg, no physical limitations, pain)? | 0 = “Very poor health”  10 = “Very good health” |
| 2 | On a scale of 0 to 10, how do you rate your mental health (eg, no feelings of anxiety, depression)? |  |

1. **Quality of life**

| **#** | **Translated Item** | **Response** |
| --- | --- | --- |
| 1 | On a scale of 0 to 10, how would you rate your current quality of life? | 0 = “Very low quality of life”  10 = “Very high quality of life” |

1. **Sociodemographic variables**

| **#** | **Translated Items** | **Response** |
| --- | --- | --- |
| 1 | Please enter your age: | Textbox |
| 2 | Please enter your gender: | 1 = Female  2 = Male  3 = Diverse |
| 3 | Please state your current marital status: | 1 = Married  2 = Living in a relationship  3 = Single  4 = Divorced/Widowed  5 = Other |
| 4 | What is your highest educational qualification? | 1 = no school degree  2 = secondary school certifcate (Hauptschule)  3 = secondary school certificate (Mittlere Reife)  4 = university entrance qualification  5 = university’s degree  6 = academic degree  7 = Other  8 = Not specified |
| 5 | Please indicate your current employment status: | Attending School/Study  Employed (part time)  Employed (full time)  Sick leave  Retirement/Pension  Not employed  Other |
| 6 | Please indicate the type of city or municipality in which you live: | 1 = Big city (from 100,000 inhabitants)  2 = Medium city (from 20,000 inhabitants)  3 = Small city (from 5,000 inhabitants)  4 = Rural village (below 5,000 inhabitants) |

1. **Covid-19 related variables**

| **#** | **Translated Items** | **Response** |
| --- | --- | --- |
| 1 | When did you receive evidence of SARS-COV2 in the nasopharyngeal swab? | Textbox |
| 2 | Was hospital treatment necessary? | 1 = Yes  2 = No |
| 3 | Did you need intensive medical care? | 1 = Yes  2 = No |
| 4 | Please select what applies to you in relation to the course of your COVID-19 disease: | 1 = “I had no symptoms.”  2 = “I had mild symptoms.”  3 = “I had moderate symptoms.”  4 = “I had severe symptoms.” |
| 5 | I currently experience my physical capacity as ... | 1 = “Still significantly limited”  2 = “Average”  3 = “Good” |
| 6 | What current symptoms are you suffering from? | - Sore throat - Cough - Shortness of breath - Headache/pain in the limbs - Body temperature above 38 °C - Olfactory and/or gustatory disturbances - Diarrhea - Other symptoms (text field) |
