# Supplementary material for: Assessing Electronic Health Literacy in Individuals With the Post–COVID-19 Condition Using the German Revised eHealth Literacy Scale: Validation Study
Source: JMIR Form Res. 2024 Apr 25;8:e52189. doi: 10.2196/52189 (PMC11082733; doi:10.2196/52189)
Supplement: Multimedia Appendix 3 [file formative_v8i1e52189_app3.docx]

| **Items** | **Factor Loadings** | |
| --- | --- | --- |
|  | **information seeking** | **information appraisal** |
| GR-eHEALS1 | .80 | - |
| GR-eHEALS2 | .83 | - |
| GR-eHEALS3 | .82 | - |
| GR-eHEALS4 | .91 | - |
| GR-eHEALS5 | - | .82 |
| GR-eHEALS6 | - | .73 |
| GR-eHEALS7 | - | .75 |
| GR-eHEALS8 | - | .82 |
