# Supplementary material for: Assessing Electronic Health Literacy in Individuals With the Post–COVID-19 Condition Using the German Revised eHealth Literacy Scale: Validation Study
Source: JMIR Form Res. 2024 Apr 25;8:e52189. doi: 10.2196/52189 (PMC11082733; doi:10.2196/52189)
Supplement: Multimedia Appendix 4 [file formative_v8i1e52189_app4.docx]

| Variable | | physical health | mental health | quality of life | internal health locus of control |
| --- | --- | --- | --- | --- | --- |
| physical health | | | | | |
|  | *r* | 1 | .40 | .69 | .31 |
|  | *P* value | - | <.001 | <.001 | <.001 |
| mental health | | | | | |
|  | *r* | .40 | 1 | .54 | .22 |
|  | *P* value | <.001 | - | <.001 | <.001 |
| quality of life | | | | | |
|  | *r* | .69 | .54 | 1 | .36 |
|  | *P* value | <.001 | <.001 | - | <.001 |
| internal health locus of control | | | | | |
|  | *r* | .31 | .22 | .36 | 1 |
|  | *P* value | <.001 | <.001 | <.001 | - |
