# Supplementary material for: Assessing Electronic Health Literacy in Individuals With the Post–COVID-19 Condition Using the German Revised eHealth Literacy Scale: Validation Study
Source: JMIR Form Res. 2024 Apr 25;8:e52189. doi: 10.2196/52189 (PMC11082733; doi:10.2196/52189)
Supplement: Multimedia Appendix 5 [file formative_v8i1e52189_app5.docx]

| **Sociodemographic  variable** | **Invariance level** | **Chi²** | **df** | **CFI** | **TLI** | **RMSEA** | **SRMR** | **ΔCFI** | **ΔChi²** | **df^a^** | ***P*^a^** |
| --- | --- | --- | --- | --- | --- | --- | --- | --- | --- | --- | --- |
| Gender | configural | 83.87 | 38 | 0.99 | 0.99 | 0.036 | 0.040 | < 0.01 | - | - | - |
|  | metric | 60.81 | 44 | 0.99 | 0.99 | 0.026 | 0.043 | < 0.01 | 2.78 | 6 | .84 |
|  | scalar | 66.74 | 50 | 0.99 | 0.99 | 0.024 | 0.045 | < 0.01 | 5.64 | 6 | .47 |
| Age | configural | 84.93 | 38 | 0.99 | 0.99 | 0.038 | 0.042 | < 0.01 | - | - | - |
|  | metric | 90.46 | 44 | 0.99 | 0.99 | 0.047 | 0.057 | < 0.01 | 11.32 | 6 | .079 |
|  | scalar | 113.71 | 50 | 0.99 | 0.99 | 0.051 | 0.062 | < 0.01 | 24.68 | 6 | < .001 |
| Educational level | configural | 84.93 | 38 | 0.99 | 0.99 | 0.038 | 0.042 | < 0.01 | - | - | - |
|  | metric | 73.09 | 44 | 0.99 | 0.99 | 0.036 | 0.047 | < 0.01 | 7.4 | 6 | .29 |
|  | scalar | 90.67 | 50 | 0.99 | 0.99 | 0.039 | 0.052 | < 0.01 | 18.43 | 6 | < .01 |

^a^ df and P refer to the Chi² difference test; CFI = Comparative Fit Index; TLI = Tucker Lewis index; RMSEA = Root Mean Square Error of Approximation; SRMR = Standardized Root Mean Square Residual; ΔCFI = change in CFI compared to the less restrictive model; ΔChi² = change in Chi² compared to the less restrictive model;

Note that the column Chi² reports robust test statistics while the ΔChi² is based on standard (not robust) test statistics
